# Coding-Complete Genome Sequences of an Omicron Subvariant (BA.5.2.20) of SARS-CoV-2

**DOI:** 10.1128/mra.00077-23

**Published:** 2023-05-24

**Authors:** Zineb Rhazzar, Mouhssine Hemlali, Marouane Melloul, Maha Ouarab, Omar Nyabi, Mostafa Elouennass, Elmostafa El Fahime, Nadia Touil, Jean-Luc Gala, Khalid Ennibi

**Affiliations:** a Cell Culture Unit, Center of Virology, Infectious and Tropical Diseases, Mohammed V Military Teaching Hospital, Rabat, Morocco; b Immunopathology Research Team (ERIP), Faculty of Medicine and Pharmacy, University Mohammed V, Rabat, Morocco; c Neuroscience and Neurogenetics Research Team, Faculty of Medicine and Pharmacy, University Mohammed V, Rabat, Morocco; d Molecular Biology and Functional Genomics Platform, National Center for Scientific and Technical Research (CNRST), Rabat, Morocco; e Center for Applied Molecular Technologies (CTMA), Institute of Clinical and Experimental Research, Université Catholique de Louvain, Brussels, Belgium; f Department of Bacteriology, Mohammed V Military Teaching Hospital, Rabat, Morocco; g Genomic Center for Human Pathologies (GENOPATH), Faculty of Medicine and Pharmacy, University Mohammed V, Rabat, Morocco; Queens College Department of Biology

## Abstract

Here, we present the complete coding sequences of two severe acute respiratory syndrome coronavirus 2 (SARS-CoV-2) strains that were recovered from a nasopharyngeal swab from a female patient and the second viral passage in cell culture. After testing, both strains were identified as BA.5.2.20, a subvariant of Omicron.

## ANNOUNCEMENT

Severe acute respiratory syndrome coronavirus 2 (SARS-CoV-2), a member of the genus *Betacoronavirus* and family *Coronaviridae*, is the causative agent of coronavirus disease 2019 (COVID-19) (1). First reported in Morocco in March 2020, it has resulted in 1.27 million cases and 16,294 deaths (2). In November 2021, the WHO announced the emergence of a new variant of concern (VOC) named Omicron (B.1.1.529) (3). We report here the coding-complete genome sequence of 2 SARS-CoV-2 isolates exhibiting low threshold cycle (*C_T_*) values. Sample 1 was a nasopharyngeal specimen collected from a 25-year-old female patient (RdRp gene *C_T_* value, 14.66), and sample 2 was recovered from the second viral passage on 293TACE2.TMPRSS2 cells (ATCC NR-55293_12-2021). These cells were modified to express TMPRSS2 and ACE2, hence increasing their permissivity to Omicron (4).

Both samples were sequenced. Viral RNAs were extracted using the NucleoSpin RNA virus isolation kit (Macherey-Nagel, Germany). Sequencing reagents were purchased from Thermo Fisher Scientific (USA). The RNA quantity and quality were evaluated using the Qubit RNA high-sensitivity (HS) assay kit; cDNA was then synthesized from a sufficient amount of RNA (1 to 10 ng) using the SuperScript VILO reverse transcriptase kit. Libraries were prepared using the Ion AmpliSeq SARS-CoV-2 research panel, according to the manufacturer’s instructions, and then were loaded into the Ion Chef instrument for automated template preparation and chip loading. Sequencing was carried out on the Ion S5 sequencer. Finally, consensus sequences were generated by mapping the reads against the Wuhan-Hu-1 reference sequence (GenBank accession number NC_045512.2) and the reference sequence of Omicron variant BA.1 (Pango lineage B.1.1.529; OL672836.1) using Unipro UGENE version 45 and a *de novo* mapping tool (UGENE version 46.0). All tools were run with default parameters unless otherwise specified.

**FIG 1:**
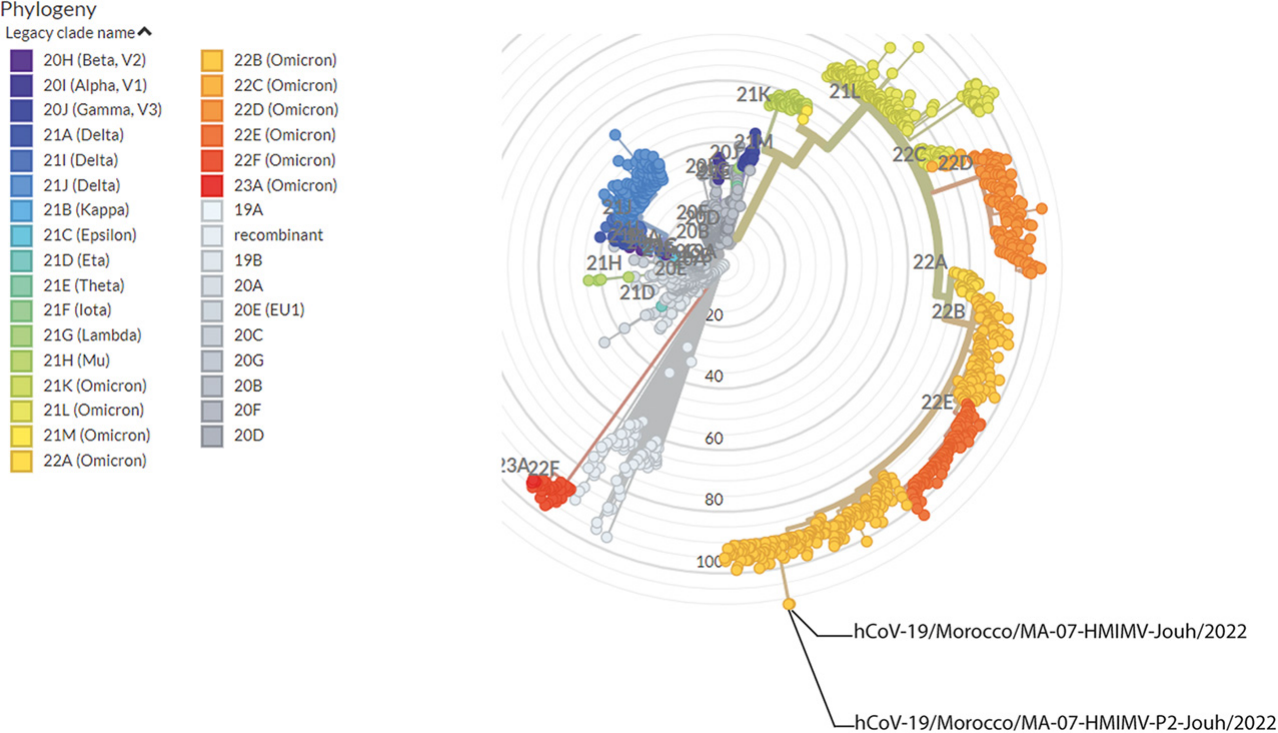
Phylogenetic tree displaying the sequenced SARS-CoV-2 strains (with reference to the global GISAID dataset), according to the clades assigned to the virus in the phylogenetic analysis using the Augur toolkit (with default settings) run by the Nextstrain server. The location of our strains, hCoV-19/Morocco/MA-07-HMIMV-Jouh/2022 (sample 1) and hCoV-19/Morocco/MA-07-HMIMV-P2-Jouh/2022 (sample 2), is marked by a large yellow circle with a red outline.

We obtained 495,971 and 654,008 reads (length, 29,903 bp; GC content, 37.92%). Phylogenetic analysis using the Pangolin web application (https://pangolin.cog-uk.io/) identified the strains as belonging to subvariant BA.5.2.20 of Omicron lineage B.1.1.529 (Fig. 1). This lineage was first reported in South Africa on 9 November 2021 (5).

The Nextclade Web application (https://clades.nextstrain.org/) was used to identify 34 identical amino acid substitutions in both samples against the Wuhan-Hu-1 reference sequence (Table 1). Both genomes had 16 amino acid changes in the spike protein, including the S-to-F change at position 371 (S371F) and S373P in the receptor binding domain (RBD) region and L452R in the receptor binding motif (RBM) region. These mutations alter the site conformations of the S protein, resulting in reduced sensitivity to neutralizing antibodies (6, 7), a decrease in treatment efficacy (8, 9), and high transmissibility (10). Other protein mutations (N, E, M, and ORF1ab) were also detected with P681H in the S1/S2 furin cleavage site, which can prompt a rapid fusion between the virus and the cell membrane, resulting in increasing viral pathogenesis (11, 12).

**TABLE 1:** Amino acid changes, all present in both hCoV-19/Morocco/MA-07-HMIMV-Jouh/2022 (sample 1) and hCoV-19/Morocco/MA-07-HMIMV-P2-Jouh/2022 (sample 2) compared to the reference strain^*a*^

Mutation location	Mutation type
S	T19I^*b*^
	A27S
	G339D
	S371F
	S373P
	K417N
	N440K
	L452R
	D614G
	H655Y
	N679K
	P681H
	N764K
	D796Y
	Q954H
	N969K
ORF1a	S135R
	T842I
	G1307S
	T1682I
	L3027F
	T3255I
	P3395H
ORF1b	R1315C
	T2163I
ORF9b	P10S
	D16G
E	T9I
M	D3N
	A63T
N	P13L
	R203K
	G204R
	S413R

a Reference strain:Wuhan-Hu-1 (GenBank accession number NC_045512.2).

b T19I, T-to-I change at position 19.

Genomic surveillance and cell culture are two complementary approaches for understanding SARS-CoV-2 evolution, as cell culture helps us understand and study the cell entry of SARS-CoV-2 variants.

### Data availability.

The SARS-CoV-2 genome sequences were submitted to the GISAID database under the identifiers EPI_ISL_16683686 and EPI_ISL_16683687 and to NCBI GenBank under the accession numbers OQ341628 and OQ341629. The reads Binary Alignement Map (BAM) were also submitted to the SRA under BioProject accession numbers PRJNA945815 and PRJNA945874.
